# The Correlation Between the Attended Deaths at Home and Medical Resources in Osaka City

**DOI:** 10.7759/cureus.44585

**Published:** 2023-09-02

**Authors:** Katsuya Nitta, Haruaki Naito, Yasuhiro Kakiuchi

**Affiliations:** 1 Department of Forensic Medicine, Kindai University, Osakasayama, JPN

**Keywords:** japan, medical resources, end-of-life care, postmortem examination, attended death at home

## Abstract

Introduction

Most people would prefer end-of-life care to be provided at home. Although Japan has tried to promote home care and end-of-life care, very few people die at home in Japan. On the other hand, deaths at home are not necessarily attended deaths at home by end-of-life care because they include many deaths, such as deaths from external causes and solitary deaths. We obtained the data on the number of postmortem examinations at home in the main areas of Osaka City and calculated the estimated number of attended deaths at home by subtracting the number of postmortem examinations at home from the number of total deaths at home. We analyzed the contribution of medical resources to end-of-life care from a forensic perspective for a closer analysis of the actual situation.

Methods

The data about the population, the number of total deaths, deaths at home, and medical resources related to home care in Osaka City was obtained from the website of a public institution in Japan. The data about the number of postmortem examinations in Osaka City was obtained from the Osaka Medical Examiner’s Office. The estimated number of attended deaths at home was calculated by subtracting postmortem examinations at home from total deaths at home. We conducted univariate and multivariate analyses between the number of medical resources and the prevalence of attended deaths at home.

Results

In the univariate analysis of the prevalence of attended deaths at home, a high positive correlation was observed in “doctors,” “total clinics,” “clinics except HCSC,” and “general beds.” A high negative correlation was observed in “long-term care beds.” In the multivariate analysis, a positive coefficient was observed in “clinics except HCSC,” and a negative one was observed in “HCSC or HCSH.”

Conclusion

The policy of shifting general clinics and hospitals to HCSC and HCSH may not be as effective for end-of-life care because the criteria do not include any restrictions on the number or use of beds. However, general clinics may have played an important role in end-of-life care, even if they were not HCSC.

## Introduction

Regarding preferences for the place to die, the majority of middle-aged and older adults and patients would prefer that end-of-life care be provided at home [1-3]. However, Japan has one of the lowest prevalence of death at home among developed countries, accounting for approximately 15% of all deaths [4-9]. Since 2006, the Ministry of Health, Labor, and Welfare (MHLW) has also promoted a shift from general clinics and hospitals to Home Care Support Clinics (HCSC) and Home Care Support Hospitals (HCSH) and established a policy to develop home care and end-of-life care [10].

However, there is an important drawback in previous studies in the world that have attempted to analyze the contribution of medical resources to home care and end-of-life care based on the prevalence of deaths at home [9,11-15]. Because deaths at home include not only attended deaths by end-of-life care but also deaths that undergo postmortem examinations, such as solitary deaths, suicides, and deaths due to external causes, it is difficult to evaluate end-of-life care based on deaths at home. In addition, the data from postmortem examinations are not publicly available, and it is difficult for researchers not associated with the police to apply for use. One study in Japan pointed out that 3%-14% of deaths at home are due to external causes such as falling, drowning, and accidental suffocation and that even if those due to external causes were eliminated, many solitary deaths due to illness would remain [16]. In particular, Japan is known to have an extremely high prevalence of solitary deaths. Even if the solitary deaths were restricted to those in which more than two days elapsed between the death and discovery, solitary deaths account for approximately 1% of total deaths and 23% of deaths at home, making it problematic to use deaths at home for the analysis of end-of-life care [17].

Most solitary deaths and deaths due to external causes require postmortem examinations by forensic doctors and doctors who work alongside the police. In this study, we obtained data on the number of postmortem examinations performed on deaths at home from the Osaka Prefectural Medical Examiner’s Office, which performs most postmortem examinations in Osaka City, the second most populous city in Japan. We estimated the number of attended deaths at home by subtracting the number of postmortem examinations from the number of deaths at home and analyzed the contribution of medical resources to end-of-life care more closely.

## Materials and methods

The number of total deaths and deaths at home in the main areas of Osaka City in 2021 was obtained from the vital statistics in Japan [4]. The number of postmortem examinations at home in 2021 was obtained from the Osaka Medical Examiner’s Office with permission. The information on population and medical resources regarding home care in each main area was obtained from the website of the Japan Medical Association [18].

As explanatory variables, the number of doctors and some types of clinics, hospitals, and hospital beds were chosen. In Japan, to apply for HCSC and HCSH, there are several criteria related to the system of doctor’s visits and nurse’s visits at any time, the number and work of the doctors who are in charge of home care, and past achievements of doctor’s visits and taking care of patients attended death. The detailed definitions of HCSC and HCSH are described in the MHLW [19].

The estimated number of attended deaths at home was calculated by subtracting postmortem examinations at home from total deaths at home [20]. The prevalence of attended deaths at home was defined as the number of attended deaths at home divided by total deaths. We conducted Pearson product-moment correlation coefficient and multiple linear regression analysis using EZR version 1.55. Referring to the previous study, the number of medical resources was standardized by 1,000 people aged 65 years and older in these analyses [12]. In the Pearson product-moment correlation coefficient, we defined the value from 0.7 to 1.0 as high positive, from 0.4 to 0.7 as medium positive, from 0.2 to 0.4 as low positive, from -0.2 to -0.4 as low negative, from -0.4 to -0.7 as medium negative, and from -0.7 to -1.0 as high negative correlation. These analyses were conducted between each medical resource and the prevalence of attended deaths at home. In the multiple linear regression analysis, the limit of explanatory variables was considered to be approximately one per two samples, referring to the report and the setting of the EZR [21]. In this study, we planned to use up to two explanatory variables in a multiple linear regression analysis. In the analysis, all variance inflator factors (VIF) were under two.

## Results

Table 1 shows the numbers and prevalence of deaths in the main areas of Osaka City in 2021. Among the main areas, the prevalence of deaths at home was 21.6%-24.6%. The prevalence of attended deaths at home was 8.8%-11.7%.

**Table 1:** The numbers and prevalence of deaths in the main areas of Osaka City

Main area	Total deaths, N (%)	Home		
		Deaths at home, N (%)	Postmortem examinations, N (%)	Attended deaths at home, N (%)
					North	7144 (100)	1755 (24.6)	917 (12.8)	838 (11.7)
South	11823 (100)	2576 (21.8)	1538 (13.0)	1038 (8.8)
East	7495 (100)	1687 (22.5)	863 (11.5)	824 (11.0)
West	5041 (100)	1090 (21.6)	607 (12.0)	483 (9.6)

Table 2 shows the numbers and prevalence of the population and the medical resources related to home care in the main areas of Osaka City.

**Table 2:** The population and the medical resources related to home care in the main areas of Osaka City HCSC: Home Care Support Clinics; HCSH: Home Care Support Hospitals

Main area	Population, N (%)		Doctors, N (%)	Clinics, N (%)			Hospitals, N (%)				HCSC or HCSH, N (%)	Hospital beds, N (%)	
	Total	≧65 years old		Total	HCSC	Except HCSC	Total	HCSH	Sub-HCSH	Except HCSH and sub-HCSH		General beds	Long-term care beds
North	697514 (100)	158337 (22.7)	2951 (100)	878 (100)	155 (17.7)	723 (82.3)	36 (100)	8 (22.2)	4 (11.1)	24 (66.7)	163 (17.8)	6532 (87.4)	943 (12.6)
South	810235 (100)	234674 (29.0)	2366 (100)	874 (100)	253 (28.9)	621 (71.1)	46 (100)	9 (19.6)	2 (4.3)	35 (76.1)	262 (28.5)	5756 (68.7)	2618 (31.3)
East	755327 (100)	170089 (22.5)	2921 (100)	1009 (100)	240 (23.8)	769 (76.2)	62 (100)	20 (32.3)	5 (8.1)	37 (59.7)	260 (24.3)	8701 (87.9)	1197 (12.1)
West	489336 (100)	113721 (23.2)	1616 (100)	456 (100)	109 (23.9)	347 (76.1)	28 (100)	4 (14.3)	9 (32.1)	15 (53.6)	113 (23.3)	4364 (81.8)	974 (18.2)

Table 3 shows the Pearson product-moment correlation coefficient between the medical resources and the prevalence of attended deaths. A high positive correlation was observed in the numbers of “doctors,” “total clinics,” “clinics except HCSC,” and “general beds.” A medium positive one was observed in “total hospitals,” “HCSH,” and “sub-HCSH.” A low positive one was observed in “HCSC” and “HCSC or HCSH.” A high negative one was observed in “long-term care beds.”

**Table 3:** The Pearson product-moment correlation coefficient between medical resources and the prevalence of attended deaths at home HCSC: Home Care Support Clinics; HCSH: Home Care Support Hospitals

	Doctors	Clinics			Hospitals				HCSC or HCSH	Hospital beds	
		Total	HCSC	Except HCSC	Total	HCSH	Sub-HCSH	Except HCSH and sub-HCSH		General beds	Long-term care beds
Correlation coefficient	0.98	0.92	0.21	0.98	0.45	0.50	0.43	-0.08	0.26	0.77	-0.97

Table 4 shows the multivariate linear regression analysis between the main medical resources related to end-of-life care and the prevalence of attended deaths at home. Regarding the prevalence of attended deaths at home, a positive coefficient was observed in the number of “clinics except for HCSC,” and a negative one was observed in “hospitals except for HCSH and sub-HCSH.”

**Table 4:** The multivariate linear regression analysis between the main medical resources related to end-of-life care and the prevalence of attended deaths at home SE: Standard Error; HCSC: Home Care Support Clinic; HCSH: Home Care Support Hospital A p-value of ≤0.05 was taken as statistically significant

	The prevalence of attended deaths at home			
	Coefficient	SE	T-value	P-value
Intercept	6.43	0.09	75.50	0.008
HCSC or HCSH	-1.37	0.08	-17.87	0.036
Clinic except HCSC	1.47	0.02	77.32	0.008

## Discussion

As shown in Table 1, at least in Osaka City, approximately half of the deaths at home underwent postmortem examinations, which makes it difficult to use the total deaths at home for the analysis of end-of-life care. The extent to which postmortem examinations account for the prevalence of deaths at home in each area depends on several factors, such as the percentage of single-person households and public safety and cannot be considered solely in terms of end-of-life care. Considering that solitary deaths tend to occur in single-person households that require medical services but do not receive them, it can be said that the prevalence of deaths at home includes many deaths that are the opposite of attended deaths.

The multivariate analysis shows that the prevalence of attended deaths at home increased by about 1.5% by a clinic except HCSC per 1,000 elderly persons and decreased by about -1.4% by a HCSC or HCSH per 1,000 elderly persons. The trend seen in previous studies in Japan was that the HCSC has a significant positive or no significant correlation with the prevalence of total deaths at home, while the HCSH has a significant negative or no significant correlation [12,15]. Despite being specific to home care and end-of-life care providers, they are not always significantly positively correlated with the prevalence of total deaths or attended deaths at home. Furthermore, the prevalence of total deaths at home in Japan has not increased since 2006, when the system of HCSC and HCSH was initiated. First, it should be noted that approximately 25% of clinics and hospitals are accredited as HCSC or HCSH [22]. However, about 10%-20% of clinics and hospitals are not HCSC or HCSH but are in charge of home care and end-of-life care [22]. Considering our result, the policy of shifting general clinics and hospitals to HCSC and HCSH has not been successful in increasing the prevalence of attended deaths at home. The main possible reason is the absence of restrictions on the number and use of beds in the criteria. Japan is one of the countries with the highest number of total beds and long-term care beds, but the problem has been neglected [23]. Since 2006, Japan's main policy has been to increase the number of home care and end-of-life care, and there are financial benefits to being approved as HCSC and HCSH. The categories of enhanced HCSC and HCSH were also created, but there are still no restrictions regarding the number and use of beds. As mentioned above, there has been little discussion on the criteria itself because no firm conclusions have been reached regarding whether HCSC and HCSH have contributed to end-of-life care. Although this study has some limitations, it showed that HCSC and HCSH may not be effective against attended deaths. Especially considering that the prevalence of deaths at home in Japan was low and had much to grow, we need to seriously discuss where the problem lies.

First, the major limitation of this study was that the sample size was insufficient because it covered only Osaka City. Thus, only two variables can be selected for the multiple linear regression analysis. This is due to the difficulty in obtaining data on the number of postmortem examinations at home because there are few big facilities for postmortem examinations and autopsies covering certain areas except Osaka City. Osaka City is the second-most populous city, but the result is not necessarily applicable to Japan. Second, in our study, "clinics except HCSC" had a positive correlation and "HCSC or HCSH" had a negative correlation. General hospitals cannot apply to HCSH if there is a clinic in the surrounding area [20]. Therefore, HCSH has a limit on increasing, while HCSC does not. Since it is uncertain whether HCSH is currently in the process of increasing, there are some problems with analyzing HCSC and HCSH at the same time. Third, the types of medical resources in this study are less detailed on home care and end-of-life care than those in the previous studies [12,15]. The available information on medical resources depends on the area. While their samples were prefectures, ours were within a city, and the information was limited. As mentioned above, the area where the data for postmortem examinations can be obtained is limited in Japan, and it is difficult to take advantage of both benefits.

The strength of this study is that the number of attended deaths was calculated from a forensic perspective, which is close to the actual situation. As mentioned above, one study attempted to calculate the number of attended deaths by subtracting the number of deaths due to external causes from the number of deaths at home [16]. However, using the data from postmortem examinations in Osaka City, we were able to exclude deaths further, such as solitary deaths due to illness. Since this kind of data is only available from police organizations and large facilities related to forensic medicine in the area, we are taking advantage of our strengths as forensic scientists.

## Conclusions

In Osaka City, approximately half of the deaths at home underwent postmortem examinations, which makes it difficult to use the deaths at home for the analysis of end-of-life care. Regarding the prevalence of attended deaths at home, “clinics except HCSC” showed a positive correlation, and “HCSC or HCSH” showed a negative correlation. The policy of shifting general clinics and hospitals to HCSC and HCSH may not be as effective for end-of-life care because the criteria do not include any restrictions on the number or use of beds. However, general clinics may have played an important role in end-of-life care, even if they were not HCSC.

## References

[1] Higginson IJ, Sen-Gupta GJ (2000). Place of care in advanced cancer: a qualitative systematic literature review of patient preferences. J Palliat Med.

[2] Cabinet Office (2023). Cabinet Office: Results of the 2012 health awareness survey of the elderly. Internet.

[3] Cabinet Office (2023). Cabinet Office: Results of the 2007 health awareness survey of the elderly. Internet.

[4] (2023). e-Stat: Vital statistics about the number of deaths in Japan in 2021. https://www.e-stat.go.jp/stat-search/files?page=1&layout=datalist&toukei=00450011&tstat=000001028897&cycle=7&year=20210&month=0&tclass1=000001053058&tclass2=000001053061&tclass3=000001053074&tclass4=000001053085&result_back=1&tclass5val=0.

[5] Dasch B, Blum K, Gude P, Bausewein C (2023). Place of death: trends over the course of a decade: a population-based study of death certificates from the years 2001 and 2011. Dtsch Arztebl Int.

[6] De Roo ML, Miccinesi G, Onwuteaka-Philipsen BD (2014). Actual and preferred place of death of home-dwelling patients in four European countries: making sense of quality indicators. PLoS One.

[7] Cross SH, Warraich HJ (2019). Changes in the place of death in the United States. N Engl J Med.

[8] (2023). Gov.UK: Statistical commentary: end of life care profiles. Public Health England.

[9] Kjellstadli C, Han L, Allore H, Flo E, Husebo BS, Hunskaar S (2019). Associations between home deaths and end-of-life nursing care trajectories for community-dwelling people: a population-based registry study. BMC Health Serv Res.

[10] (2023). Ministry of Health, Labour and Welfare of Japan: Health and medical services. https://www.mhlw.go.jp/english/wp/wp-hw3/02.html.

[11] Morioka N, Tomio J, Seto T, Yumoto Y, Ogata Y, Kobayashi Y (2018). Association between local-level resources for home care and home deaths: A nationwide spatial analysis in Japan. PLoS One.

[12] Ikeda T, Tsuboya T (2021). Place of death and density of homecare resources: a nationwide study in Japan. Ann Geriatr Med Res.

[13] Miyashita M, Shirai Y, Sanjo M, Hasada T, Sato K, Misawa T (2007). Association of medical, social indicators, and the prevalence of deaths at home by prefecture in 2004. Journal of Health and Welfare Statistics.

[14] Alawneh A, Anshasi H (2021). Place of death for patients treated at a tertiary cancer center in Jordan. Support Care Cancer.

[15] Tarasawa K, Fujimori K, Ogata T, Chiba H (2023). Associations of death at home with medical resources and medical activities in cancer patients: a nationwide study using Japanese national database. Ann Geriatr Med Res.

[16] Taniguchi Y, Watanabe T, Midorikawa H, Tachikawa H, Tamiya N (2020). The prevalence of deaths at home from external causes among total deaths at home by municipality nationwide. Journal of Health and Welfare Statistics.

[17] Kakiuchi Y, Nagao R, Ochiai E, Kakimoto Y, Osawa M (2019). A descriptive study of solitary death in Yokohama City. Environ Health Prev Med.

[18] (2023). JMAP: Statistics on the population and medical resources of 24 wards in Osaka City. https://jmap.jp/cities/detail/medical_area/2708.

[19] (2023). Ministry of Health, Labour and Welfare: Explanatory materials on the revision of medical service fees in 2022. https://www.mhlw.go.jp/stf/seisakunitsuite/bunya/0000196352_00008.html.

[20] Kakiuchi Y, Nagao R, Ochiai E, Kakimoto Y, Osawa M (2019). The importance of the rate of pure "attended deaths at home" for objective outcome indicator for assessing the prevalence of home care in Japan. Environ Health Prev Med.

[21] Austin PC, Steyerberg EW (2015). The number of subjects per variable required in linear regression analyses. J Clin Epidemiol.

[22] (2023). Ministry of Health, Labour and Welfare: About the promotion of home care. Internet.

[23] (2023). OECD: Health at a glance 2021. https://www.oecd.org/health/health-at-a-glance/.

